# The membrane trafficking and functionality of the K^+^-Cl^−^ co-transporter KCC2 is regulated by TGF-β2

**DOI:** 10.1242/jcs.189860

**Published:** 2016-09-15

**Authors:** Eleni Roussa, Jan Manuel Speer, Ilona Chudotvorova, Shokoufeh Khakipoor, Sergei Smirnov, Claudio Rivera, Kerstin Krieglstein

**Affiliations:** 1 Institute of Anatomy and Cell Biology, Department of Molecular Embryology, Faculty of Medicine, University of Freiburg, Albertstrasse 17, Freiburg D-79104, Germany; 2 Institute of Anatomy and Cell Biology, Department of Neuroanatomy, Faculty of Medicine, University of Freiburg, Albertstrasse 17, Freiburg D-79104, Germany; 3 Institute of Biotechnology, University of Helsinki, Viikinkaari 9, Helsinki FIN-00014, Finland

**Keywords:** KCC2, CREB, Growth factor, Neuronal development, Rab11b

## Abstract

Functional activation of the neuronal K^+^-Cl^−^ co-transporter KCC2 (also known as SLC12A5) is a prerequisite for shifting GABA_A_ responses from depolarizing to hyperpolarizing during development. Here, we introduce transforming growth factor β2 (TGF-β2) as a new regulator of KCC2 membrane trafficking and functional activation. TGF-β2 controls membrane trafficking, surface expression and activity of KCC2 in developing and mature mouse primary hippocampal neurons, as determined by immunoblotting, immunofluorescence, biotinylation of surface proteins and KCC2-mediated Cl^−^ extrusion. We also identify the signaling pathway from TGF-β2 to cAMP-response-element-binding protein (CREB) and Ras-associated binding protein 11b (Rab11b) as the underlying mechanism for TGF-β2-mediated KCC2 trafficking and functional activation. TGF-β2 increases colocalization and interaction of KCC2 with Rab11b, as determined by 3D stimulated emission depletion (STED) microscopy and co-immunoprecipitation, respectively, induces CREB phosphorylation, and enhances Rab11b gene expression. Loss of function of either *CREB1* or *Rab11b* suppressed TGF-β2-dependent KCC2 trafficking, surface expression and functionality. Thus, TGF-β2 is a new regulatory factor for KCC2 functional activation and membrane trafficking, and a putative indispensable molecular determinant for the developmental shift of GABAergic transmission.

## Abstract (precis)

**Summary:** TGF-β2 promotes K^+^-Cl^−^-co-transporter KCC2 trafficking and functional activation in hippocampal neurons through a TGF-β2, CREB and Rab11b signaling pathway.

## INTRODUCTION

Regulation of KCC2 (also known as SLC12A5), the neuron-specific electroneutral K^+^-Cl^−^ co-transporter, is crucial for development and maturation of GABAergic neurotransmission. In immature central nervous system (CNS) neurons, γ-aminobutyric acid (GABA) produces depolarizing postsynaptic potentials, which are likely involved in stabilizing synapses during development (Ben-Ari, 2002; Blaesse et al., 2009). GABA-mediated depolarization is sustained through a high expression of NKCC1 (also known as SLC12A2), a cation-chloride co-transporter that mediates intracellular Cl^−^ accumulation above its electrochemical equilibrium. During maturation of most central neurons, however, expression of the Cl^−^ extruder KCC2 is upregulated resulting in an intracellular Cl^−^ concentration below its electrochemical equilibrium, thereby shifting GABA_A_ responses from depolarizing to hyperpolarizing (Rivera et al., 1999). The mechanisms underlying KCC2 regulation have been extensively investigated but still are not fully understood. Brain-derived neurotrophic factor (BDNF) has been shown to upregulate KCC2 expression in immature neurons, whereas in mature neurons BDNF–TrkB signaling results in an activity-dependent decrease of KCC2 expression (Rivera et al., 2002, 2004). Accordingly, KCC2 expression is decreased in early postnatal TrkB-deficient mice (Carmona et al., 2006). However, recent observations have challenged the dominant role of BDNF in the developmental upregulation of KCC2 by demonstrating that BDNF, although a potent regulator, is not a necessary molecular determinant for the required KCC2 upregulation during development (Puskarjov et al., 2015). Indeed, other trophic factors, such as neurturin, also possess the ability to regulate this transporter (Ludwig et al., 2011).

KCC2 functionality is achieved not only through transcriptional control, but also through regulation of KCC2 membrane trafficking, integration and stabilization in the membrane. KCC2 protein (de)phosphorylation is thought to be a crucial regulatory mechanism for KCC2 surface expression, surface stability and trafficking. Apparently, the functional consequences of phosphorylation on KCC2 depend on the specific residue (reviewed by Kahle et al., 2013). Along this line, PKC-dependent phosphorylation of KCC2 at S940 increases its cell surface expression and promotes KCC2 membrane stability in cultured hippocampal neurons (Lee et al., 2007), whereas WNK-kinase-dependent phosphorylation at T906 and T1007 inhibits KCC2 transport function (Rinehart et al., 2009). In contrast, Src-mediated Y903 and/or Y1087 phosphorylation regulates the membrane trafficking of KCC2 and decreases the cell surface stability of KCC2 by enhancing its lysosomal degradation (Lee et al., 2010). Besides (de)phosphorylation, other molecular pathways that affect KCC2 membrane trafficking in immature neurons include TrkB and 5-HT_2A_ serotonin receptors (Khirug et al., 2005, 2010; Bos et al., 2013), whereas for mature neurons the molecular mechanisms underlying KCC2 membrane trafficking need to be elucidated.

TGF-βs are multifunctional, extracellular signaling molecules that exert a wide range of biological responses on different cell types, including cells of the nervous system. In the CNS, TGF-βs are required for cell fate decisions (Farkas et al., 2003; Lu et al., 2005; Roussa et al., 2006, 2008) and regulate neuronal survival and apoptosis during nervous system development (Dünker and Krieglstein, 2000; Krieglstein et al., 2000). Furthermore, an impact of TGF-βs on synaptogenesis, neural network function and neuronal plasticity has been shown (Krieglstein et al., 2011). Early studies have documented effects such as a TGF-β1-induced long-term facilitation in *Aplysia* sensory-motor synapses and increase in neuronal excitability (Zhang et al., 1997; Chin et al., 1999), effects mediated through activation of MAPK signaling (Chin et al., 2006) and modulation of synapsin distribution by phosphorylation (Chin et al., 2002). TGF-β2 has been also identified as a local modulator of the neuromuscular junction through the control of presynaptic quantal size (Fong et al., 2010). The TGF-β isoforms, namely TGF-β1, TGF-β2 and TGF-β3, exhibit a distinct spatial and temporal expression pattern and, although targeted mutations of individual TGF-β genes are lethal, the phenotypes are distinct and isoform-specific. *Tgfb2^−/−^* mutants die at birth due to congenital cyanosis, yet cardiovascular and pulmonary causes of lethality have been excluded (Sanford et al., 1997). Interestingly, impaired synaptic transmission of spontaneous GABAergic or glycinergic, and glutamatergic postsynaptic currents in the respiratory control area, the pre-Bötzinger complex (preBötC), has been demonstrated (Heupel et al., 2008).

In the present study, we show that TGF-β2 can control KCC2 trafficking and activity in mature hippocampal neurons. We also identify the signaling pathway TGF-β2–CREB–Rab11b as the underlying mechanism for TGF-β2-mediated KCC2 trafficking and activity. Our results introduce TGF-β2 as a new regulator of KCC2 functionality and as putative crucial determinant for the developmental shift of GABAergic transmission.

## RESULTS

### KCC2 membrane trafficking is controlled by TGF-β2

**Fig. 1.**
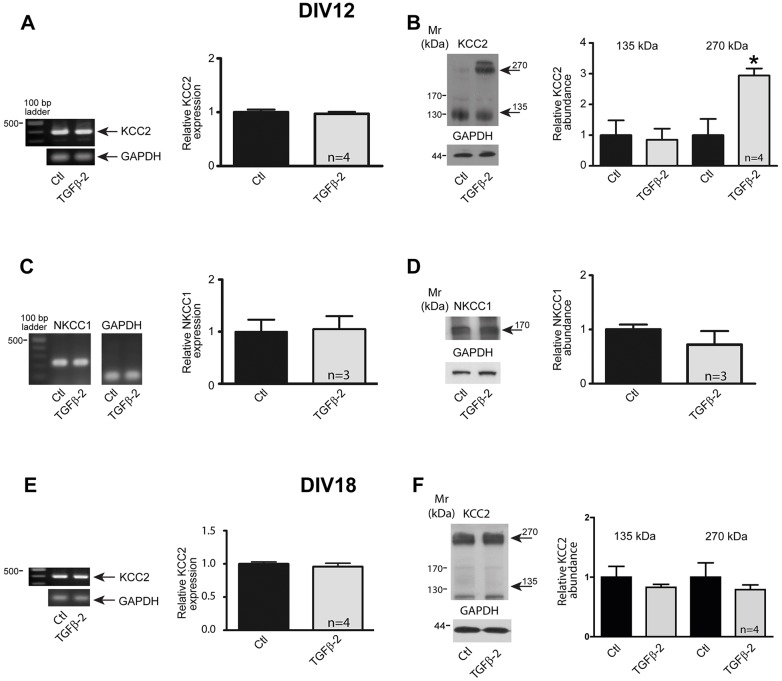
**Regulation of KCC2 in neurons at different developmental stages by TGF-β2.** (A) Developing (DIV12) cultured mouse hippocampal neurons were treated with 2 ng/ml TGF-β2 for 60 min. KCC2 transcript (397 bp) expression was normalized to GAPDH by semi-quantitative RT-PCR. Data are given as fold changes compared to control. (B) DIV12 cultured neurons were treated with TGF-β2 before immunoblotting with anti-KCC2 antibody. Arrows point to the ∼135 kDa and ∼270 kDa KCC2 bands. The ratio of KCC2:GAPDH immunoreactivity was determined. *P=0.016 relative to control as assessed by an unpaired *t*-test (*n*=4). (C,D) DIV12 cultured mouse hippocampal neurons were treated with 2 ng/ml TGF-β2 for 60 min followed by by semi-quantitative RT-PCR analysis (C) or immunoblotting (D). The ratio of the NKCC1:GAPDH transcript expression (in control set to 1) and the ratio of the NKCC1:GAPDH immunoreactivity were then determined. (E) Semi-quantitative RT-PCR analysis in more mature (DIV18) cultured mouse hippocampal neurons treated with 2 ng/ml TGF-β2 for 60 min, (*n*=4). (F) Immunoblot analysis for ∼135 kDa (arrow) and ∼270 kDa (arrow) KCC2 protein in cultures of more mature hippocampal neurons upon TGF-β2 treatment. Data are given as mean±s.e.m. for the indicated number of experiments.

The mechanisms regulating KCC2 membrane expression and activity are complicated but appear to involve signaling induced by trophic factors (Rivera et al., 2002, 2004; Ludwig et al., 2011). With this in mind, we first addressed the question of whether TGF-β2 regulates KCC2 mRNA and protein expression during neuronal development. Hippocampal neurons were isolated at embryonic day (E)18.5 and cultured for 12 or 18 days *in vitro* (DIV). As shown in Fig. 1A, KCC2 transcript expression (397 bp) was detectable in neurons cultured for 12 days (Rivera et al., 2002; Ludwig et al., 2003) and a 60-min pulse of TGF-β2 did not further increase KCC2 transcript expression (Fig. 1A). However, TGF-β2 treatment of the cultures induced a ∼270-kDa KCC2 band (Fig. 1B). In contrast, NKCC1 (Fiumelli and Woodin, 2007) transcript (235 bp; Fig. 1C) and protein expression (Fig. 1D) remained unchanged following a 60-min pulse of TGF-β2. In more mature neurons, cultured for 18 days (Dotti et al., 1988), application of TGF-β2 for 60 min had no effect on KCC2 transcript (Fig. 1E) and protein expression (Fig. 1F).

**Fig. 2.**
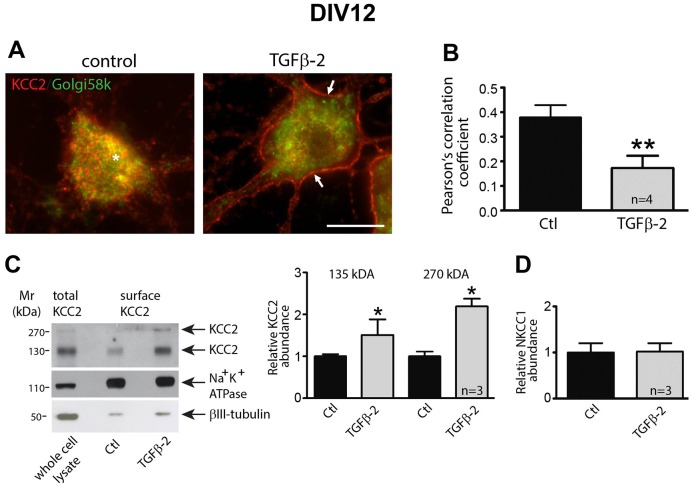
**KCC2 membrane trafficking in hippocampal neurons is controlled by TGF-β2.** (A) Mouse hippocampal neurons cultured for 12 days were treated for 60 min with 2 ng/ml TGF-β2, followed by immunolabeling for KCC2 (red) and the Golgi marker Golgi58k (green). The asterisk indicates intracellular KCC2–Golgi58k colocalization and the arrows point to membrane KCC2 labeling. Scale bar: 10 µm. (B) Colocalization of KCC2 and Golgi58k was quantified by determination of the Pearson's correlation coefficient. **P=0.0015 compared to the control condition (unpaired Student's *t*-test). (C,D) Cultured neurons were treated with TGF-β2 for 60 min followed by biotinylation of surface proteins. The ratio of ∼135 kDa surface KCC2:total KCC2, ∼270 kDa surface KCC2:total KCC2 (C), and of surface NKCC1:total NKCC1 (D) in untreated (Ctl) and TGF-β2-treated cultures were then determined and presented relative to values for controls (set to 1). *P=0.014 compared to the control condition (unpaired Student's *t*-test). Data are given as mean±s.e.m. from three or four independent experiments as indicated.

We next investigated the cellular localization of KCC2 in response to the TGF-β2 treatment, using DIV12 and DIV18 cultures. As shown in Fig. 2A, KCC2 immunoreactivity under control conditions at DIV12 was predominantly associated with small intracellular vesicles which partly colocalized with the Golgi marker Golgi58k (asterisk). A 60-min TGF-β2 pulse cleared the majority of these KCC2 immunoreactive vesicles from intracellular stores and shifted immunoreactivity to the cell membrane (arrows). The quantification shown in Fig. 2B revealed that TGF-β2 treatment significantly reduced KCC2–Golgi58K colocalization (*P*=0.0015, *n*=4), Moreover, immunoblotting following biotinylation of cell surface proteins showed a significant increase for both the KCC2∼135-kDa and ∼270-kDa band (*P*=0.014, *n*=3, Fig. 2C), but not for NKCC1 (Fig. 2D) upon TGF-β2 treatment at DIV12.

**Fig. 3.**
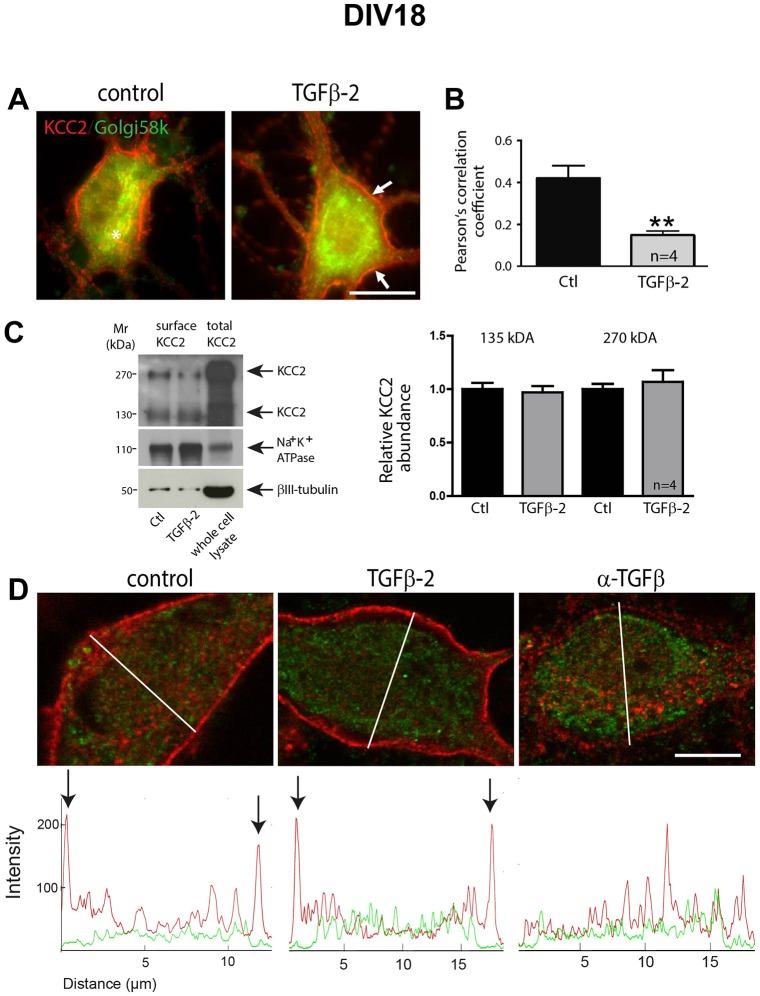
**TGF-β2 is required for KCC2 trafficking to the membrane in mature hippocampal neurons.** (A) Double immunofluorescence for KCC2 (red) and the Golgi marker Golgi58k (green) in DIV18 hippocampal cultures following application of TGF-β2 (2 ng/ml) for 60 min. Arrows and asterisk indicate membrane and intracellular KCC2, respectively. Scale bar: 10 µm. (B) Trafficking of KCC2 was assessed by determination of colocalization between KCC2 and Golgi58k using the Pearson's correlation coefficient. After TGF-β2 treatment, the Pearson's correlation coefficient for KCC2 and Golgi58k was significantly decreased. Data are given as mean±s.e.m. from four independent cultures and experiments. **P<0.01 (unpaired Student's t-test). (C) Cultured neurons at DIV18 were treated with TGF-β2 for 60 min followed by biotinylation of surface proteins. The ratio of ∼135 kDa surface KCC2:total KCC2, ∼270 kDa surface KCC2:total KCC2 in untreated (Ctl) and TGF-β2-treated cultures were then determined and presented relative to values for controls (set to 1). Error bars represent s.e.m. (D) Double immunofluorescence for KCC2 (red) and the Golgi marker Golgi58k (green) in DIV18 hippocampal cultures following application of TGF-β2 (2 ng/ml) or anti-TGF-β (10 µg/ml) at DIV12 and DIV15. Line scans illustrate the KCC2 (in red) and Golgi58K (in green) distribution profile, and arrows indicate peaks of KCC2 immunofluorescence (representative line scans from three independent experiments).

In 18-day-old cultures (Fig. 3A,B), following TGF-β2 treatment for 60 min, KCC2–Golgi58K colocalization was significantly reduced (*P*=0.0084, *n*=4). Immunoblotting following biotinylation of cell surface proteins (Fig. 3C) showed no significant differences for both the KCC2∼135-kDa and ∼270-kDa band upon TGF-β2 treatment at DIV18. Given that TGF-β is endogenously expressed in hippocampal neurons, we treated the cultures either with TGF-β2 or with anti-TGF-β, a TGF-β function-blocking antibody, at DIV12 and DIV15, and assessed KCC2 immunoreactivity at DIV18 (Fig. 3D). Representative line scans from cells under each experimental condition illustrate the distribution profile for KCC2 (red) and Golgi58k (green) immunoreactivity. Peaks of KCC2 labeling were detected in the periphery of cell bodies of control and TGF-β2-treated cells, suggesting membrane labeling, whereas after neutralizing endogenous TGF-β, KCC2 was exclusively intracellularly distributed.

Taken together, these results provide the first evidence for a TGF-β2-dependent trafficking of KCC2 from the Golgi to the cell membrane in developing and mature neurons. These changes might induce a functional activation of KCC2.

### Functional expression of KCC2 is controlled by TGF-β2

**Fig. 4.**
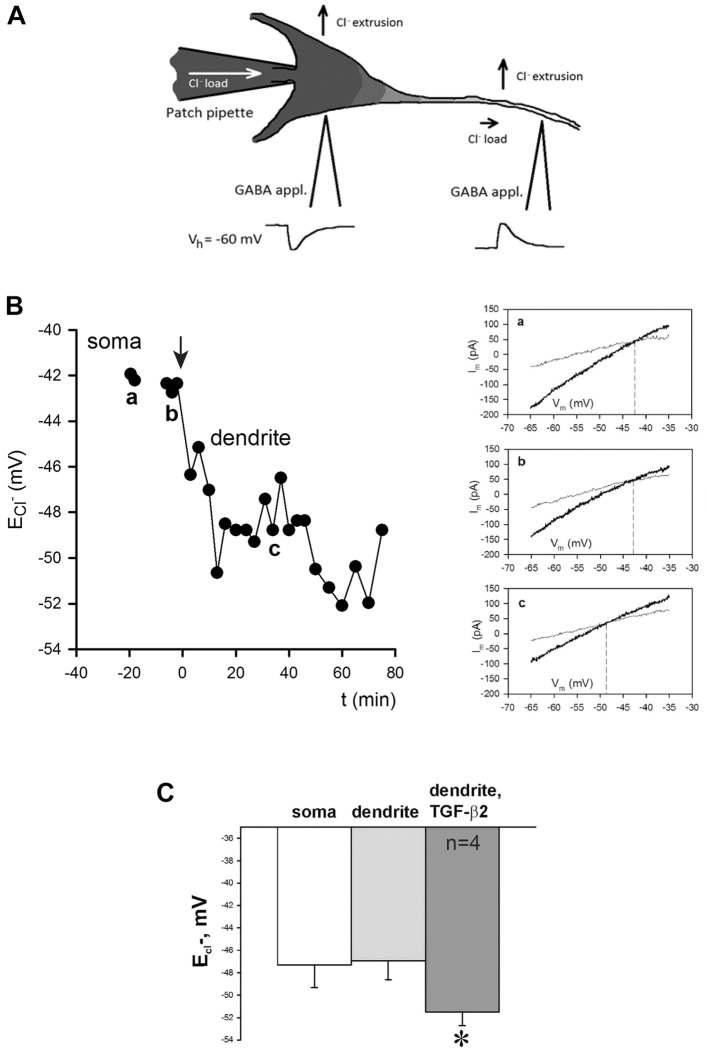
**Treatment with TGF-β2 augments extrusion of intracellular Cl^−^ from cultured hippocampal neurons.** (A) Schematic representation of the experimental paradigm for assessing changes in dendritic chloride extrusion. GABA is locally applied at the soma and at a primary dendrite (100 µm distal from the soma) of a neuron recorded in whole-cell voltage clamp mode with a pipette containing a slight load of chloride. The reversal potential in for GABA is estimated in the two positions. (B) Timecourse of Cl^−^ reversal potential measured in soma (a) and dendrite (b,c) of a cultured (DIV 10) hippocampal neuron. Onset of TGF-β2 application corresponds to time point zero. In this particular neuron, almost no somatodendritic Cl^−^ gradient was observed before exposure to TGF-β2 as the difference in E_Cl−_ measured in soma (a) and in dendrite (b) was almost zero. After application of TGF-β2, dendritic E_Cl−_ and therefore somatodendritic Cl^−^ gradient became negative (c). The insets are example traces of voltage ramps before (light gray) and during local application of GABA (dark gray) at the soma (a) and the dendrites (b,c). The intercept between these traces gives an estimation of the GABA_A_ reversal potential at the specific location. b and a are example traces before and after application of TGF-β. (C) Quantification of dendritic E_Cl−_ in control and TGF-β2-treated neurons. *P<0.05 compared to the control (unpaired Student's *t*-test). Error bars represent s.e.m. from four independent experiments. Data used in this panel include only those experiments where no statistically significant difference between somatic and dendritic E_Cl−_ measured in the same cell was observed prior to TGF-β2 application (Fig. S1).

TGF-β2-dependent translocation of KCC2 to the cell membrane in developing neurons does not necessarily imply functional integration of KCC2. We therefore investigated whether TGF-β2 could activate KCC2 by means of altering efficacy of KCC2-mediated Cl^−^ extrusion in hippocampal neurons. To do this, whole-cell patch-clamp recordings were taken from primary hippocampal neurons at DIV10. Under a constant Cl^−^ load mediated by a somatic patch pipette, transport-active KCC2 generates a negative somatodendritic electrochemical Cl^−^ gradient (Khirug et al., 2005) (Fig. 4A). We determined E_Cl−_ at the soma and at proximal dendrites and we observed declining somatodendritic Cl^−^ gradients after treatment with exogenous TGF-β2. Fig. 4B and Fig. S1 illustrate examples of neurons with distinct KCC2 activity. In the neuron illustrated in Fig. 4A, soma E_Cl−_ was −43 mV close to the calculated value (−50 mV) expected following a constant load with 19 mM [Cl^−^] through the patch pipette. E_Cl−_ in the dendrite was similar to that of the soma, demonstrating that, in this particular neuron, KCC2 is not yet active. Treatment with TGF-β2 (arrow) caused a negative shift in somatodendritic Cl^−^ gradients, demonstrating effective KCC2-mediated Cl^−^ extrusion activity. In more mature neurons (see Fig. S1) soma E_Cl−_, was also close to −50 mV, as expected from the chloride load, but dendrite E_Cl−_ was more negative (−55 mV), due to activated KCC2. Exposure to TGF-β2 (arrow) caused a small decline in the somatodendritic Cl^−^ gradient. Somatic and dendritic E_Cl−_ in less mature neurons prior to TGF-β2 treatment were −47.32±2.01 and −46.94±1.71 mV, respectively, and the somatodendritic Cl^−^ gradient was 0.38±0.41 mV (mean±s.e.m.; Fig. 4C). TGF-β2 treatment of these neurons induced a negative shift in the dendritic E_Cl−_ of −4.58±0.62 mV (*n*=4; dendritic E_Cl−_ being −51.51±1.23 mV, *P*<0.01 when a paired *t*-test was applied for dendritic E_Cl−_ prior to and during exposure to TGF-β2; Fig. 4C) when measured as an average over the time window of 20–40 min after onset of TGF-β2 application. The above data demonstrate that TGF-β2 can activate KCC2, indicated by increased cellular Cl^−^ extrusion.

### TGF-β2-induced CREB phosphorylation affects KCC2 trafficking

**Fig. 5.**
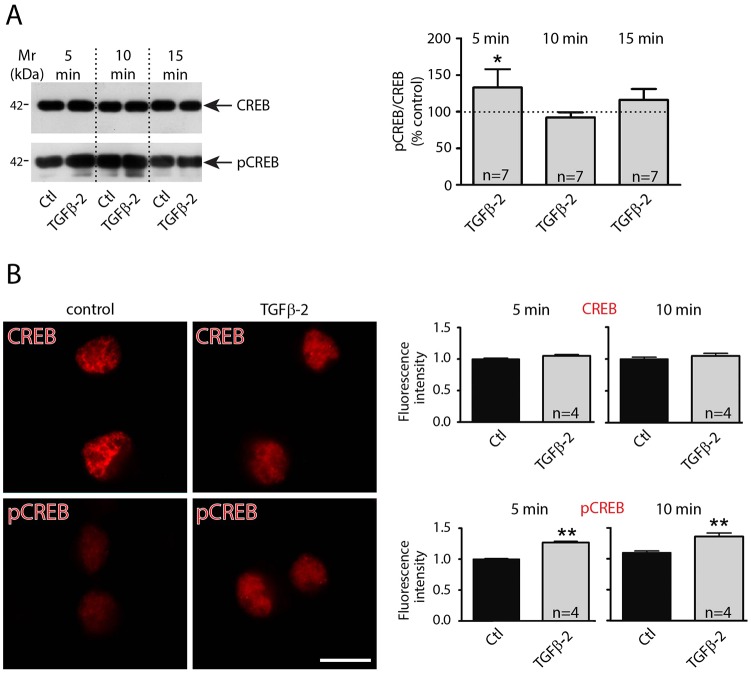
**TGF-β2 activates CREB by increasing its phosphorylation.** (A) Western blot analysis of total and of phosphorylated CREB in cultured hippocampal neurons (DIV12) treated with 2 ng/ml TGF-β2 for either 5, 10 or 15 min (dotted line represents values for control). (B) Immunofluorescence for CREB1 and pCREB of mouse hippocampal cultures (DIV12) under control conditions and following treatment with TGF-β2 for 10 min. Scale bar: 10 µm. Quantification of relative CREB1 and phospho-CREB fluorescence intensity following application of TGF-β2 for 5 and 10 min (images are representative out of four experiments). Data are shown as mean±s.e.m. for seven or four experiments as indicated. *P<0.05, **P<0.01 (unpaired Student's *t*-test).

**Fig. 6.**
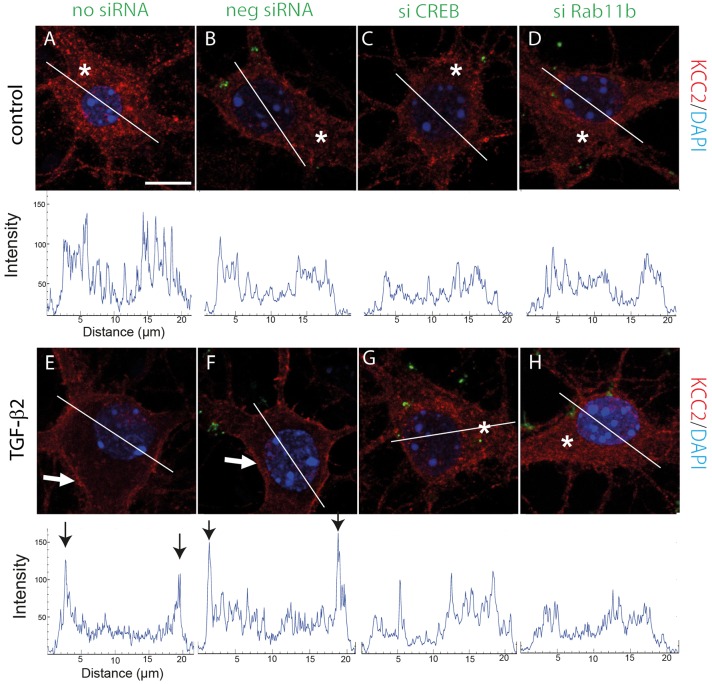
**Loss-of-function of either *CREB* or *Rab11b* impairs TGF-β2-mediated KCC2 trafficking to the plasma membrane.** Immunofluorescence for KCC2 in primary mouse hippocampal neurons transiently transfected with control negative siRNA (neg siRNA, green in B and F), with specific siRNA against *CREB1* (si CREB, green in C and G) or with specific siRNA against *Rab11b* (si Rab11b, green in D and H). Cells shown in A and E represent non-transfected neurons. At 24 h following transfection cells were treated with 2 ng/ml TGF-β2 for 60 min. Nuclei were labeled with DAPI (blue). Scale bar: 10 µm. Asterisks indicate cytosolic KCC2 localization, white arrows point to plasma membrane labeling. The KCC2 distribution profile was visualized by line scans for KCC2 immunofluorescence. Black arrows indicate peaks of KCC2 immunoreactivity. Representative images and line scans are from four independent experiments.

We next addressed putative mechanisms underlying TGF-β2-mediated trafficking of KCC2 to the membrane. In rat hippocampal neurons TGF-β2 has been shown to induce CREB phosphorylation (Fukushima et al., 2007). We therefore investigated protein abundance and cellular distribution of total CREB1 and of phosphorylated CREB in primary cultured hippocampal neurons (DIV12) and in neurons exposed to recombinant TGF-β2 for 5, 10 and 15 min. Immunoblot analysis (Fig. 5A) revealed that treatment with TGF-β2 for 5 min significantly upregulated CREB phosphorylation, compared to the untreated controls (*P*<0.05, *n*=7; unpaired Student's *t*-test). Immunofluorescence (Fig. 5B) confirmed these data at the cellular level (*P*<0.01). We next investigated whether TGF-β2-induced CREB phosphorylation promotes KCC2 trafficking to the cell membrane. Hippocampal neurons were transfected either with negative small interfering RNA (siRNA) or with Alexa-Fluor-488-labeled specific siRNA against *CREB1*, as previously described (Oehlke et al., 2012). Cells that had been exposed to the transfection reagent only served as controls (no siRNA). After determination of the efficiency of *CREB1* transcript knockdown following transfection (Fig. S2), cells were treated with TGF-β2 for 60 min and cellular localization of KCC2 (red) was assessed. Fig. 6 illustrates the KCC2 distribution pattern together with the corresponding line scans for randomly depicted neurons for the experimental conditions used. In controls, localization of KCC2 was similar in non-transfected cells (Fig. 6A), in cells transfected with control negative siRNA (Fig. 6B) and in cells transfected with specific *CREB1* siRNA (Fig. 6C). In these experiments, KCC2 consistently revealed intracellular localization (asterisks and line scan). After treatment with TGF-β2 (Fig. 6E–G), KCC2 localization was shifted to the plasma membrane in both non-transfected cells (Fig. 6E) and in cells transfected with control negative siRNA (Fig. 6F) (arrows). As shown in representative line scans from cells for each experimental condition, peaks for KCC2 immunolabeling (arrows) are present at the periphery of neuronal cell bodies, suggesting labeling of the plasma membrane. In contrast, KCC2 remained localized within the cytosol in cells transfected with specific *CREB1* siRNA (Fig. 6G; asterisk and respective line scan). Thus, interfering with CREB prevented TGF-β2-mediated KCC2 translocation from intracellular pools to the plasma membrane.

### Rab11b mediates TGF-β2-dependent KCC2 trafficking

**Fig. 7.**
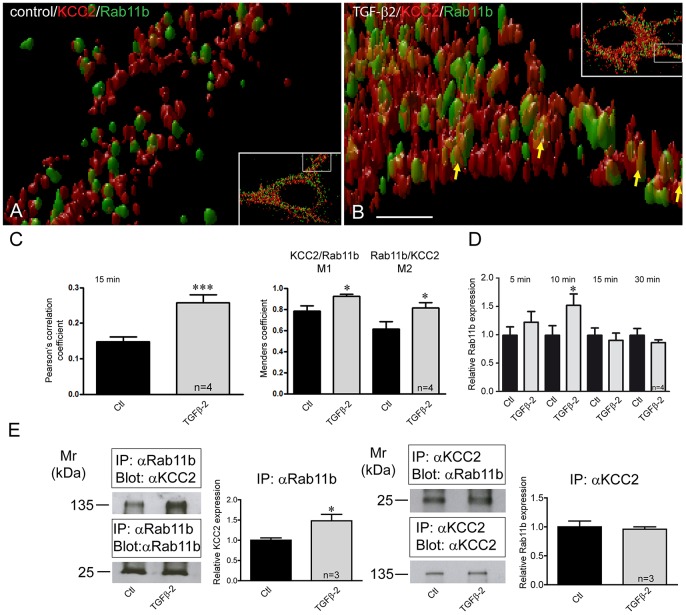
**Rab11b colocalizes and interacts with KCC2 and its expression depends on TGF-β2.** (A,B) 3D STED images illustrating part of dendrite of an untreated control neuron (inset in A) and a TGF-β2-treated neuron (inset in B) were acquired. High magnification images correspond to the white-boxed area of the inset. Scale bar: 500 nm. (C) Pearson's correlation coefficient and Manders' coefficients for KCC2 and Rab11b were calculated and were significantly increased in neurons treated with TGF-β2 for 15 min. ***P=0.0005 for the Pearson's correlation coefficient, and *P=0.015 and *P=0.025 for M1 and M2, respectively (unpaired Student's *t*-test). (D) DIV12 cultured mouse hippocampal neurons were treated for 5–30 min with 2 ng/ml TGF-β2, followed by quantitative RT-PCR analysis for *Rab11b* expression. *P=0.033 for relative *Rab11b* expression after TGF-β2 application (unpaired Student's *t*-test). (E) Interaction of KCC2 with Rab11b in controls and TGF-β2-treated DIV12 hippocampal neurons. Antibodies against Rab11b were able to immunoprecipitate (IP) KCC2 expressed in control hippocampal neurons, as detected by immunoblotting with KCC2 antibody. Antibodies against KCC2 were also able to immunoprecipitate Rab11b expressed in control hippocampal neurons, as detected by immunoblotting with Rab11b antibody. The ratio of ∼135 kDa KCC2:input Rab11b, ∼25 kDa Rab11b:input KCC2 in untreated (Ctl) and TGF-β2-treated cultures were then determined and presented relative to values for controls (set at 1). The amount of co-immunoprecipitated KCC2 was significantly increased in TGF-β2-treated neurons, compared to the untreated controls (*P=0.04, unpaired Student's *t*-test). Data are given as mean±s.e.m. from three or four independent experiments as indicated.

Rab GTPases are established players of vesicular trafficking (Hutagalung and Novick, 2011). Rab11b has been shown to mediate vesicular transport of transmembrane proteins (Silvis et al., 2009; Oehlke et al., 2011; Butterworth et al., 2012). In addition, Rab11b has been found in other cellular systems to be under transcriptional control of CREB (Zhang et al., 2005). We therefore explored whether Rab11b was a target of TGF-β2 in the transport of KCC2-carrying vesicles to the neuronal plasma membrane. Using 3D stimulated emission depletion (STED) microscopy, we first investigated colocalization of KCC2 (red) with Rab11b (green) in control and TGF-β2 treated DIV12 hippocampal neurons. Representative pictures at low and high magnification are shown in Fig. 7. KCC2 colocalization with Rab11b in dendrites of control neurons (Fig. 7A) was very low, and yellow KCC2–Rab11b clusters are hardly detectable. In contrast, after TGF-β2 treatment (Fig. 7B), colocalization of KCC2 with Rab11b in dendrites was considerably increased, represented by increased appearance of KCC2–Rab11b yellow clusters (arrows). Quantification of the colocalization revealed that Pearson's correlation coefficient was significantly increased in cells treated with TGF-β2 for 15 min (r=0.148±0.01 and r=0.257±0.07, for controls and TGF-β2-treated neurons, respectively; *P*=0.0005, Fig. 7C). Manders' coefficients M1 and M2, representing colocalization of KCC2 with Rab11b and Rab11b with KCC2, respectively, were also significantly increased in TGF-β2 treated cells [M1, 0.783±0.05 (controls) and 0.921±0.02 (treated), *P*=0.015; M, 0.614±0.07 (controls) and 0.814±0.05 (treated), *P*=0.025, Student's *t*-test]. In addition, the size of the KCC2–Rab11b clusters was apparently increased in TGF-β2-treated cells. We next examined regulation of Rab11b expression in DIV12 cultured primary hippocampal neurons and in neurons exposed to TGF-β2 for 5 min to 30 min. Quantitative real-time PCR analysis (Fig. 7D) revealed that treatment with TGF-β2 significantly upregulated Rab11b expression with a peak at 10 min.

Confirmation that KCC2 and Rab11b colocalization is significantly increased in TGF-β2-treated hippocampal neurons does not necessarily imply that these proteins interact with each other. To test for a physical association of Rab11b and KCC2, co-immunoprecipitation experiments were performed. As illustrated in Fig. 7E, Rab11b co-immunoprecipitated KCC2 in controls, and TGF-β2 treatment significantly increased the amount of immunoprecipitated KCC2 (1.00±0.06 and 1.48±0.16 for controls and TGF-β2-treated cells, respectively; *P*<0.05, *n*=3, Student's *t*-test). Vice versa, immunoprecipitation of KCC2 co-immunoprecipitated Rab11b as well, however, without differences between controls (1.00±0.10 fold) and TGF-β2-treated cultures (0.96±0.04 fold; *n*=3). The efficiency of association between Rab11b and KCC2 is further determined by detection of the respective input in the co-immunoprecipitation. These data demonstrate that Rab11b and KCC2 can indeed be associated with each other in mouse hippocampal neurons and that TGF-β2 might increase their interaction.

To investigate whether TGF-β2-dependent KCC2 translocation to the plasma membrane is mediated by Rab11b we knocked down Rab11b expression, as described previously (Oehlke et al., 2011), by transfecting the neurons with Alexa-Fluor-488-labeled specific siRNA against *Rab11b* (see Fig. S2). Cells that had been exposed to the transfection reagent only served as controls. Similar to the results obtained after knockdown of *CREB1*, cells transfected with siRNA specific for *Rab11b* and treated with TGF-β2, revealed intracellular KCC2 localization (Fig. 6H, asterisk and corresponding line scan). Thus, interfering with Rab11b precluded TGF-β2-mediated KCC2 translocation from intracellular pools to the plasma membrane.

To analyze whether Rab11b acts downstream of CREB, we determined Rab11b transcript and protein expression following knockdown of *CREB1*. As shown in Fig. S2, transfection of cells with specific siRNA against *CREB1* downregulated Rab11b mRNA and protein. These data support the notion that Rab11b acts downstream of CREB and mediates TGF-β2-dependent KCC2 trafficking.

### Rab11b mediates TGF-β2-dependent KCC2 functional expression

**Fig. 8.**
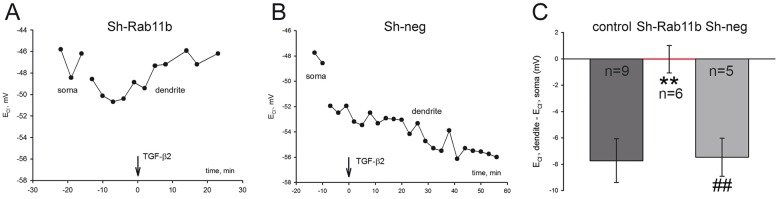
**Rab11b mediates TGF-β2-dependent KCC2 functional expression.** (A) Somatodendritic Cl^−^ gradients measured in cultured neurons (DIV10–15) transfected with Sh-Rab11b (*n*=6 independent experiments) and treated with TGF-β2. (B) Somatodendritic Cl^−^ gradients measured in cultured neurons (DIV10-15) transfected with Sh-neg (*n*=5 independent experiments). Control cells (*n*=9) include those characterized in Fig. 4. (C) Quantification of E_GABA_ gradient after TGF-β2 application. **P<0.01 compared to the controls, ^##^*P*<0.01 compared to Sh-Rab11b (unpaired Student's *t*-test). Statistical analysis for the cells transfected with Sh-Rab11b includes only those experiments where the ‘external control’ was positive, i.e. non-transfected cells from the same coverslip showed a clear difference in somatic and dendritic E_Cl−_. Data are shown as mean±s.e.m. for the indicated number of experiments.

To investigate whether the identified molecular pathway TGF-β2–CREB–Rab11b is the mechanism underlying TGF-β2-depedent KCC2 activation, we measured somatodendritic Cl^−^ gradients in cultured neurons transfected with short hairpin RNA against Rab11b (sh-Rab11b) and with negative control shRNA (Sh-neg) and treated with TGF-β2 (Fig. 8). In cells transfected with Sh-neg, treatment with TGF-β2 (arrow) caused a negative shift in somatodendritic Cl^−^ gradients, demonstrating effective KCC2-mediated Cl^−^ extrusion activity (Fig. 8B). In contrast, in cells transfected with Sh-Rab11b (Fig. 8A) application of TGF-β2 had no effect on the somatodendritic Cl^−^ gradient. The TGF-β2-induced shift in dendritic E_Cl-_ (control) was −5.49±0.8 mV (*n*=9) and the somatodendritic gradient in the presence of TGF-β2 was measured −7.26±2.21 mV (*n*=5) for the neurons transfected with Sh negative and −0.03±1.04 mV (*n*=6) for neurons transfected with Sh-Rab11b (mean±s.e.m.). These data clearly demonstrate that Rab11b mediates TGF-β2-dependent functional expression of KCC2.

Taken together, these results provide evidence that TGF-β2-dependent KCC2 trafficking and activity to the plasma membrane is mediated by a signaling cascade that involves CREB and Rab11b.

## DISCUSSION

In the present study, we show for the first time that trafficking, membrane expression and activity of the neuron-specific K^+^-Cl^−^ co-transporter KCC2, a key element for the ‘developmental shift’ from depolarizing to hyperpolarizing GABAergic responses (Rivera et al., 1999), can be regulated by TGF-β2.

KCC2 functionality has previously been shown to be regulated by transcriptional control, alternative splicing, trafficking and post-translational modifications (Blaesse et al., 2009). Meanwhile, KCC2 has been identified as a crucial molecular player not only during development but also that acts in a transport-independent mode to promote dendritic spine formation (Li et al., 2007; Gauvain et al., 2011; Fiumelli et al., 2013; Puskarjov et al., 2014; Llano et al., 2015). Moreover, impaired synaptic inhibition due to decreased KCC2 function has been demonstrated in many pathophysiological conditions, including epilepsy, spasticity and chronic pain (Boulenguez et al., 2010; Puskarjov et al., 2012; Gagnon et al., 2013). According to the current view, developmental upregulation of KCC2 reduces the depolarizing action of GABA; however, this can be resumed post-traumatically, where neurons likely acquire an immature state (reviewed in Kaila et al., 2014). Interestingly, during the past few years, several studies have highlighted that the fast functional modulation of KCC2 during physiological and pathophysiological conditions is achieved through post-translational events rather than regulation of the transporter at the transcriptional level (Puskarjov et al., 2012; Zhou et al., 2012).

Although some work has shown that the developmental regulation of KCC2 can be affected by growth factors the mechanisms underlying regulation of KCC2 *in vivo* are far from being understood. Previous experimental evidence has suggested that KCC2 is transcriptionally regulated by BDNF signaling through a complex signaling cascade (Rivera et al., 2002, 2004) but recent observations have demonstrated that in *Bdnf^−/−^* mice developmental upregulation of KCC2 protein and functionality was comparable to that in the wild type (Puskarjov et al., 2015). In contrast, BDNF–TrkB signaling was necessary for seizure-induced post-translational functional activation of KCC2. Indeed, during development, KCC2 can also be transcriptionally regulated by other trophic factors such as neurturin (Ludwig et al., 2011) and insulin-like growth factor (Kelsch et al., 2001). In our previous work, we have shown impaired GABAergic postsynaptic currents in neurons of the preBötC of *Tgfb2^−/−^* mice (Heupel et al., 2008). We hypothesize that TGF-β2 is required for the establishment of a neuronal network by regulating functional expression of KCC2.

### TGF-β2 regulates KCC2 membrane trafficking

Our results show that TGF-β2 mode of action on KCC2 apparently differs from that reported for BDNF, neurtrurin (Ludwig et al., 2011) and insulin-like growth factor (Kelsch et al., 2001), which are all capable of upregulating KCC2 mRNA and protein expression in neurons that are not fully developed. Treatment of hippocampal differentiating (after 12 days *in vitro*) neurons with TGF-β2 for 60 min caused a shift in KCC2 immunoreactivity to the periphery of the neurons, decreased KCC2–Golgi58k colocalization and an increase in the amount of surface KCC2. Collectively, this argues in favor of TGF-β2-mediated translocation of KCC2 from intracellular pools to the plasma membrane (Fig. 2). To our knowledge, this is the first demonstration of growth-factor-dependent KCC2 membrane trafficking*.* Moreover, TGF-β2 treatment of hippocampal neurons significantly increased the amount of a ∼270-kDa band, likely representing oligomeric KCC2 protein. Previous results indicate that oligomerization is involved in the functional activation of KCC2 and are in agreement with the potentiating effect of TGF-β2 on the efficacy of chloride extrusion, as discussed below. However, the question of whether oligomerization of KCC2 is required for transport activity has not yet been definitively answered (for a review, see Hartmann and Nothwang, 2015). DIV18 neurons already express KCC2 in the membrane, and treatment with exogenous TGF-β2 decreased KCC2–Golgi58k colocalization (Fig. 3B), without altering the amount of surface KCC2 (Fig. 3C). The effect of exogenous TGF-β2 on KCC2 trafficking at DIV18 is not detectable due to endogenous TGF-β expression, as shown in Fig. 3D. The requirement for TGF-β for KCC2 trafficking and membrane expression was demonstrated after neutralizing endogenously expressed TGF-β from DIV12 to DIV18. In these neurons, KCC2 had not reached the membrane at DIV18, highlighting the biological significance of TGF-β.

Our data also show that the TGF-β2 effect is specific for KCC2, given that gene expression, protein abundance and trafficking of NKCC1 (Fiumelli and Woodin, 2007) are independent of TGF-β2 (Fig. 1). In contrast, BDNF downregulates KCC2 transcript and protein in an activity-dependent manner in differentiating and mature hippocampal neurons (Rivera et al., 2002, 2004).

Obviously, the mere presence of plasmalemmal KCC2 protein in a neuron does not necessarily imply that it is functionally active. Proof that TGF-β2-induced KCC2 translocation to and incorporation into the plasma membrane renders KCC2 functional was provided by assessing the efficacy of neuronal Cl^−^ extrusion following application of TGF-β2 (Fig. 4). Interestingly, the potency of exogenous TGF-β2 to regulate KCC2 activity differed depending whether neurons had already established a KCC2-dependent Cl^−^ gradient (more mature neurons) or not (less mature neurons) (Fig. 4B; Fig. S1), matching the biochemical data in Figs 1 and 2. In neurons that were not mature with inactive KCC2 (Fig. 4B), TGF-β2 initiated KCC2-mediated Cl^−^ extrusion, a prerequisite for the ontogenetic change in GABA_A_-mediated responses from depolarizing to hyperpolarizing. In mature neurons with abundant functional KCC2 (Fig. S1), application of TGF-β2 showed a reduced potentiation of KCC2 activity probably reflecting that KCC2 in these neurons was already working at nearly maximal efficiency. Interestingly, the Cl^−^ gradient elicited by TGF-β2 in developing neurons reached a level close to the one found in mature neurons. These data highlight a distinct mode of action of TGF-β2 on KCC2 during maturation of neuronal networks by regulating its trafficking and KCC2-mediated Cl^−^ transport efficacy.

KCC2 membrane expression has been reported to be regulated by multiple post translational mechanisms including (de)phosphorylation (reviewed in Kahle et al., 2013), oligomerization (Blaesse et al., 2006) and cleavage by proteases (Puskarjov et al., 2012). Moreover, interaction of KCC2 with the kainate receptor subunit GluK2 (Mahadevan et al., 2014) and its auxiliary subunit Neto2 (Ivakine et al., 2013), as well as the adhesion molecule neuroligin-2 (Sun et al., 2013) might also regulate KCC2 surface expression. However, Neto2-deficient mice have a comparable reduction of total KCC2 protein and surface KCC2 abundance, suggesting that Neto2 might be required for KCC2 biogenesis and total protein stability rather than membrane trafficking (Mahadevan et al., 2015). Similarly, knockdown of neuroligin-2 in mouse cortical neurons reduces total KCC2 levels, an effect accompanied by decreased KCC2 membrane expression and a delayed GABA functional switch (Sun et al., 2013). By contrast, subunits of kainate-type glutamate receptors, such as GluK1 and GluK2, are required for KCC2 oligomerization and surface expression (Mahadevan et al., 2014). In spinal motoneurons, activation of 5-HT_2A_ serotonin receptors increases surface KCC2 after spinal cord injury (Bos et al., 2013). In the present study, the KCC2 mRNA levels were not changed after TGF-β2 treatment. However, TGF-β2 treatment significantly increased a ∼270 kDa band without having any significant effect on the total ∼135 kDa KCC2 protein, suggesting a more complicated multisite TGF-β2-dependent post-translational mechanism. Thus, the results of the present study differ from previous reports, our data introduce a molecular determinant acting predominantly on KCC2 trafficking. The growth factor TGF-β2 might regulate surface KCC2 expression and functionality with no net change in total KCC2 mRNA levels.

### CREB–Rab11b signaling underlies TGF-β2-dependent KCC2 trafficking

Phosphorylation has been proposed as a regulatory mechanism for KCC2 trafficking. PKC-dependent phosphorylation of KCC2 (S940) increases its cell surface expression, whereas Src-mediated phosphorylation (Y903 and/or Y1087), by regulating KCC2 membrane trafficking, decreases its cell surface stability (Lee et al., 2010). In the present study we have identified a new signaling pathway, from TGF-β2 to phosphorylated CREB and Rab11b, as the underlying mechanism for TGF-β2-mediated KCC2 trafficking and functional expression. Several lines of experimental evidence support this view: (1) TGF-β2 induced phosphorylation of CREB at S133 (Fig. 5) and increased Rab11b transcription in cultured mouse hippocampal neurons (Fig. 7); (2) TGF-β2 treatment increased KCC2–Rab11b colocalization (Fig. 7C) and their interaction (Fig. 7E); (3) either *CREB1* or *Rab11b* knockdown significantly impaired TGF-β2-induced KCC2 trafficking (Fig. 6); (4) knockdown of *CREB1* downregulated Rab11b transcript and protein (Fig. S2); and (5) knockdown of Rab11b abolished TGF-β2-mediated KCC2 activity by means of KCC2-mediated Cl^−^ transport efficacy (Fig. 8). TGF-β-dependent CREB phosphorylation and its physiological significance have been studied in many non-neural paradigms, including epithelial-mesenchymal transition, tumor growth inhibition (Yang et al., 2013) and fibronectin expression in mesangial cells (Peng et al., 2008). In contrast, little is known on the impact of TGF-β-dependent CREB phosphorylation in neurons. TGF-β1 enhances CREB phosphorylation in *Aplysia* sensory neurons in a MAPK-dependent manner (Chin et al., 2006), and TGF-β2 induces CREB phosphorylation in rat hippocampal neurons (Fukushima et al., 2007). The latter observation has been postulated as the mechanism underlying TGF-β2-dependent modulation of synaptic efficacy and plasticity, however, the causative link is missing. The present study, for the first time, deciphers a putative biological role of TGF-β2-dependent CREB phosphorylation in neurons. Our data propose a scenario, in which TGF-β2 could activate CREB by inducing its phosphorylation, which in turn induces Rab11b expression. Rab11b acting downstream of CREB mediates TGF-β2-dependent KCC2 trafficking and incorporation in the neuronal plasma membrane, ultimately leading to activation of KCC2. Rab11b differentially regulates the trafficking of distinct cargo, impacts upon plasma membrane expression of several proteins and is an established regulator of endosomal recycling (Hutagalung and Novick, 2011). It regulates degradation and decreases the amount of surface L-type Cav1.2 channels in cardiomyocytes (Best et al., 2011), whereas it increases surface expression of ENaC in renal cortical collecting duct cells (Butterworth et al., 2012), of CFTR in intestinal epithelial cells (Silvis et al., 2009) and of V-ATPase in salivary duct cells (Oehlke et al., 2011). In contrast, data related to actions of Rab11b in neurons are scarce. Rab11b is moderately enriched with the synaptic vesicle fraction of rat hippocampal neurons (Pavlos et al., 2010), and present in secretory vesicles of PC12 cells (Khvotchen et al., 2003). Our results show that TGF-β2 treatment leads to KCC2 enrichment after co-immunoprecipitation with Rab11b, indicating increased KCC2–Rab11b interaction to facilitate KCC2 trafficking to the membrane. However, the co-immunoprecipitated KCC2 is only a fraction of total KCC2, a pool of KCC2 that does not interact with Rab11b is still present. The latter fraction is included when immunoprecipitation for KCC2 is performed. This fact, together with the result that TGF-β2 treatment increases *Rab11b* expression but not KCC2 expression, likely explains the lack of enrichment of Rab11b after co-immunoprecipitation with KCC2 (Fig. 7E). Taken together, our results provide the first evidence for a crucial physiological role of Rab11b in neurons, by regulating trafficking of KCC2 to the membrane.

In summary, we introduce TGF-β2 as a new regulator of the neuronal KCC2 membrane trafficking, membrane expression and activity and provide mechanistic insight by identifying CREB and Rab11b as molecular determinants underlying TGF-β2-dependent KCC2 trafficking and activity. We propose an overall requirement for TGF-β2 for the developmental shift of GABAergic transmission and the development of a functional neuronal network.

## MATERIALS AND METHODS

### Animals

All protocols were carried out in accordance with German ethical guidelines for laboratory animals and approved by the Institutional Animal Care and Use Committee of the University of Freiburg (authorizations: X-10/27S and X-10/08S). Adult C57BL/6N mice of either sex were maintained on a 12-h-dark–12-h-light cycle with food and water *ad libitum*. Mice were killed by cervical dislocation and all efforts were made to minimize suffering.

### Primary cultures of mouse E18.5 hippocampal neurons

Hippocampal neurons were isolated from C57Bl6 mice at embryonic day (E)18.5 of gestation, as described previously (Lacmann et al., 2007). Cultures were treated with human recombinant TGF-β2 (2 ng/ml; R&D Systems) for 5, 10, 15, 30 and 60 min or with a pan-TGF-β antibody (a function-blocking anti-TGF-β1, -β2 and -β3 antibody; 10 µg/ml; cat. no. MAB1835, R&D Systems). At day *in vitro* (DIV)12, or DIV18 cells were processed for RT-PCR, immunoblotting or immunocytochemistry.

### Efficacy of KCC2-mediated Cl^−^ extrusion

KCC2-mediated Cl^−^ extrusion in hippocampal neurons was determined as described previously (Khirug et al., 2005). The assay of neuronal Cl^−^ extrusion is based on imposing a somatic chloride load through a whole-cell patch-clamp electrode and measuring the somato-dendritic gradient of the reversal potential of GABA_A_ receptor-mediated current responses (E_GABA_) induced along the dendrite by local iontophoretic application of GABA. Somatic recordings in immature cultured hippocampal neurons (DIV 10–14) were performed in standard extracellular solution at room temperature in the whole-cell voltage-clamp configuration using a patch-clamp amplifier, according to Khirug et al. (2005). For local iontophoretic application of GABA, brief (100 ms) positive current pulses (30–100 nA) were delivered by a sharp micropipette (100–200 MOhm when filled with 250 mM GABA in 250 mM HCl). Iontophoretic GABA injections were given not more often than once in 2 min. Constant negative current of −4 nA was applied to the micropipette in order to compensate for the passive leak of GABA. GABA was applied at the soma and at the dendrite (∼100 μm from the soma) of a given neuron. NKCC1 was blocked throughout the experiments with 10 μM bumetanide, action potentials with 1 μM TTX and GABA_B_ receptors with 1 μM CGP 55845 (Tocris Bioscience). Under these conditions, the somatodendritic gradient of E_GABA_ provides a quantitative estimate of the efficacy of KCC2-mediated Cl^−^ extrusion.

### Immunocytochemistry

Cells were fixed with methanol for 20 min at −20°C or with 4% paraformaldehyde (PFA) for 30 min and washed three times with PBS for 5 min. For double immunofluorescence, cells were treated for 15 min with 1% bovine serum albumin (BSA), followed by incubation with primary antibodies [anti-KCC2 at 1:1500 (C2366, Sigma Aldrich, Seelze, Germany), anti-Golgi58K at 1:75 (G 2404, Clone 58K-9, Sigma Aldrich, Seelze, Germany), anti-CREB at 1:500 (48H2, New England Biolabs, Frankfurt/Main, Germany), anti-phospho-CREB 1:50 (87G3, New England Biolabs, Frankfurt/Main, Germany), anti-Rab11b 1:250 (BO2P, Abnova, Taipei city, Taiwan)] overnight at 4°C. Cells were incubated with goat anti-rabbit-IgG coupled to Alexa Fluor 568 (1:500) and goat anti-mouse-IgG coupled to Alexa Fluor 488 (1:200) (Jackson Immuno Research, Suffolk, UK) for 1 h at room temperature. Cells were washed with PBS, coverslips mounted with Vectashield and viewed either with a Zeiss Axioplan2 fluorescence microscope with ApoTome module or with a Leica confocal SP8 microscope (Wetzlar, Germany).

### Immunoblotting

Primary hippocampal neurons were washed with ice-cold homogenizing buffer containing (in mM) 280 mannitol, 10 HEPES, 10 KCl, 1 MgCl_2_, adjusted to pH 7.0 and a protease inhibitor cocktail (10 µM leupeptin, 2 mM benzamidine and 0.1 mM Pefabloc^®^SC), scraped off the culture flasks with a rubber policeman, pelleted by centrifugation at 250 ***g*** for 5 min and resuspended in homogenization buffer. Homogenization was performed by sonication. Protein concentration was determined according to Bradford (1976) and samples were processed for immunoblotting. Electrophoresis and blotting procedures were performed as previously described (Brandes et al., 2007). Blots were incubated with primary antibody overnight at dilution 1:5000 for KCC2, 1:4000 for α1 subunit of Na^+^/K^+^-ATPase (05-369, Upstate/Millipore, Schwalbach, Germany), 1:2000 for NKCC1 (AB3560P, Upstate/Millipore, Schwalbach, Germany) and β-III-tubulin (Developmental Studies Hybridoma Bank, IA), 1:1000 for CREB and phosphorylated (p)CREB, and 1:10,000 for GAPDH [ab8245 (6C5), Abcam, Cambridge, UK]. After incubation with secondary antibodies (Amersham, GE Healthcare, Freiburg, Germany), blots were developed in enhanced chemiluminescence reagents and signals were visualized on X-ray film. Subsequently, films were scanned using a flat-bed scanner and the signal ratios for KCC2:GAPDH, NKCC1:GAPDH, KCC2:Na^+^/K^+^-ATPase and pCREB:CREB was quantified densitometrically for controls and TGF-β2-treated cells. The signal ratios for KCC2:GAPDH, NKCC1:GAPDH, KCC2:Na^+^/K^+^-ATPase, pCREB:CREB for untreated cells was set to 1. Relative protein abundance (signal ratio of treated cells/signal ratio of untreated cells, i.e. fold change) of the protein of interest was then plotted.

### Surface biotinylation

Hippocampal cultures were subjected to control or experimental conditions (application of recombinant TGF-β2) and then kept on ice. Isolation of cell surface proteins was performed using the Pierce^®^ (Thermo Fisher Scientific) cell surface protein isolation kit following the manufacturer's instructions. Proteins were then processed for immunoblotting with antibody against KCC2, NKCC1 and Na^+^/K^+^-ATPase, as described above.

### Immunoprecipitation

Immunoprecipitation was performed as described previously (Oehlke et al., 2011). Hippocampal neurons at DIV12 were scrapped off a Petri dish into 500 µl non-denaturing lysis buffer [50 mM Tris-HCl pH 7.4, 300 mM NaCl, 5 mM EDTA, 1%Triton X-100, Complete^TM^ proteinase cocktail (Roche Diagnostics, Mannheim, Germany) and 0.1 mM Pefabloc^®^SC]. Cells were pelleted by centrifugation at 250 ***g*** for 5 min and resuspended in non-denaturing lysis buffer. Efficient homogenization was assured by sonication. Protein concentration was determined according to Bradford (1976). Total protein (500 µg) was mixed with 75 µl protein-A–Sepharose beads (Invitrogen, Carlsbad, CA; 1:1 in immunoprecipitation buffer, i.e. non-denaturing lysis buffer containing 0.1% Triton X-100) and incubated overnight at 4°C with agitation. After centrifugation for 5 min at 2300 ***g*** to remove proteins non-specifically bound to protein-A–Sepharose, the precleared cell lysate was added to antibody-conjugated beads and incubated overnight at 4°C with permanent agitation. To prepare antibody-conjugated beads, 3 µg of antibody (KCC2 or Rab11b) and 20 µl 1% BSA in immunoprecipitation buffer were added to 75 µl of protein-A–Sepharose beads and incubated overnight at 4°C. The beads were spun down for 5 min at 2300 ***g*** and the precleared cell lysate was added as described above. Subsequently, the beads were spun down, washed three times in immunoprecipitation buffer and the supernatant was discarded. Bound proteins and their interaction partners were resuspended in 30 µl 6× Laemmli buffer (62.5 mM Tris-HCl pH 6.8, 2% SDS, 10% glycine, 5% β-mercaptoethanol, 0.001% Bromophenol Blue), heated for 5 min at 95°C and immediately cooled down on ice, and processed by SDS-PAGE.

### RT-PCR

Total RNA was isolated from hippocampal cells using the Qiagen RNeasy kit (Qiagen) according to the manufacturer's instructions, and reverse transcribed as previously described (Brandes et al., 2007). For detection of the transcripts, the following protocol was used: denaturation at 95°C for 5 min followed by 35 cycles of PCR amplification performed under the following conditions: denaturation at 95°C for 30 s, annealing at the appropriate temperature according to the primer pairs for 45 s, and elongation at 72°C for 60 s. Final extension at 72°C for 10 min was terminated by rapid cooling to 4°C. PCR products were analyzed by agarose gel electrophoresis. Subsequently, the signal ratios for KCC2:GAPDH and NKCC1:GAPDH were densitometrically analyzed. For PCR, the following primers were used: *GAPDH* (Genbank accession number: NM_008084.3), forward, 5′-CGGCCGCATCTTCTTGTG-3′ nucleotides (nt 196–213); reverse, 5′-TGACCAGGCGCCCAATAC-3′ (nt 289–272); for *KCC2* (Genbank accession number: NM_020333.2), forward, 5′-CTCAACAACCTGACGGACTG-3′ (nt 419–438), reverse, 5′-GCAGAAGGACTCCATGATGCCTGCG-3′ (nt 816–797); for *NKCC1* (Genbank accession number NM009194.3), forward, 5′-CATGGTGTCAGGATTTGCAC (nt 1893–1912), reverse, 5′-CGTTCAATTCAGCAATCAGG-3′ (nt 2128–2109).

### Quantitative real-time PCR

Isolation of total RNA from mouse E18.5 primary hippocampal cultures and subsequent quantitative real-time PCR was performed as described previously (Rickmann et al., 2007). Primers and probes were validated by analysis of a standard curve in a template dissociation curve. Real-time PCR was performed according to the manufacturer's instructions (BioRad, Munich, Germany). Cycle conditions: denaturation at 95°C for 10 min, and 40 cycles of PCR amplification at 95°C for 30 s and at the appropriate temperature according to the primer pair for 30 s and elongation at 72°C for 1 min. For qRT-PCR, the following primers were used: *CREB* (Genebank accession number NM_001037726.1), forward: 5′-GCCTCTGGTGATGTACAAACATACC-3′ (nt 794–818), reverse, 5′-GGGAGGACGCCATAACAACTC-3′ (nt 875–855); for *Rab11b* (Genebank accession number NM_008997.3), forward, 5′-GAAGCAAATCGCTGACCGTG-3′ (nt 758–777), reverse, 5′-GCTTGTTGGGTCTCTGTCCA-3′ (nt 858–839); and for *GAPDH* (Genebank accession number NM_001289726.1), forward, 5′-TGACGTGCCGCCTGGAGAAA-3′ (nt820-839), reverse, 5′-AGTGTAGCCCAAGATGCCCTTCAG-3′ (nt 917–894).

All PCRs were performed in triplicate on a MyiQ Optic I Cycler (Biorad) The mean±s.d. of the Ct values for Rab11b, CREB, and GAPDH were determined and analyzed for statistical significance. For documentation of the data shown in Fig. S2, relative mRNA levels were calculated using the comparative C_t_ method (2^−ΔΔCt^).

### Transient transfection of mouse primary hippocampal cultures

Mouse primary hippocampal neurons grown on coverslips or on six-well plates were transiently transfected with 100 ng Alexa-Fluor-488-labeled siRNA specifically targeting mouse *Rab11b* mRNA, Alexa-Fluor-488-labeled siRNA specifically targeting *CREB1* mRNA (purchased from Qiagen) or with control (negative) siRNA, a sequence that reveals no similarity with any known mammalian gene, labeled with Alexa Fluor 488 (AllStars Negative Control siRNA, Qiagen), as previously described (Oehlke et al., 2011, 2012). Cells solely exposed to the transfection reagent were considered as controls. Cells were treated with 2 ng/ml TGF-β2 for 60 min at 24 h after transfection, and were harvested and processed for either RT-PCR or immunocytochemistry. Efficacy of Rab11b and CREB transcript and protein knockdown was quantified using quantitative real-time PCR, as described above, and Rab11b and CREB immunolabeling, respectively. For functional experiments, primary cultures were treated with 1 µg/ml shRNA (SureSilencing shRNA Plasmid for Rat Rab11b from SABIOSCIENCES (KR43269G GFP carrying plasmid).

### Image acquisition and analysis

Images were acquired with a Leica TCS SP8 confocal microscope using a 20×0.75HC PL APO immersion objective lens (to acquire images to determine CREB and pCREB immunofluorescence intensity following TGF-β2 treatment at different time points) and a CS2 63×1.40 oil objective lens (to acquire images used for calculation of KCC2 fluorescence intensity after knocking down either *Rab11b* or *CREB*). Within each experiment, confocal microscope settings (laser power, detector gain and amplifier offset) were kept the same for all scans in which protein expression was compared. Slide labels were covered and only revealed after data collection. *z*-stacks of five or six optical sections with a step size of 0.5 μm were taken for at least five separate fields of view for each condition to ensure random image collection. Maximum intensity projections were created from the *z*-stacks. To quantify nuclear CREB and pCREB protein expression ImageJ (NIH) was used to measure the average intensity within the nucleus. After quantification, data were normalized to the mean of controls (non-treated neurons) in every experiment. Representative images in each figure were processed identically.

### Image acquisition using STED microscopy and analysis

For analysis of colocalization of KCC2 and Rab11b, images were acquired with Leica TCS SP8 gated stimulated emission depletion (STED 3×) microscopy using a HCX PLAPO 100×1.40 oil objective lens. Samples were prepared according to Leica microsystems quick guide with slight modifications. Briefly, primary hippocampal neurons were plated on 0.17-mm thick coverglass (Harvard apparatus, 64-0713) and maintained for 12 DIV. After treatment with 2 ng/ml TGF-β2 for 1 h, neurons were fixed with 4% PFA for 15 min and washed three times with PBS for 5 min. Cells were blocked with 2% BSA in PBS for 1 h and subsequently, incubated with mouse monoclonal KCC2 (1:150) and rabbit polyclonal Rab11b (1:200) antibody overnight at 4°C. The following day, neurons were incubated with goat anti-mouse IgG coupled to Alexa Fluor 532 (1:100) and goat anti-rabbit-IgG coupled to tetramethylrhodamine (TRITC) (1:100) for 1 h at room temperature. Cells were washed with PBS, and coverslips were mounted with Prolong Gold (Molecular Probes).

Within each experiment, identical settings for laser power, STED power and gate were used to acquire images. The wavelength of the laser was 660 nm and was adjusted to 50% of power. z-stacks of five or six optical sections with a step size of 0.21 μm were de-convolved using Huygens Software. Colocalization of KCC2 with Rab11b was assessed by analysis of Pearson's correlation coefficient and Mander's colocalization coefficient. The whole cell soma of each condition was used for quantification. Manders' M1 and M2 coefficients are defined separately for each channel so that they measure the portion of the intensity in each channel that coincide with some intensity in the other channel. Pearson's r-values were calculated for all planes of the *z*-stack of each picture, essentially calculating values for a 3D image. Pictures were visualized with Huygens Surface renderer.

### Statistics

No statistical methods were used to pre-determine sample sizes. We used GraphPad Prism 5 software for statistical analysis. Statistical significance was assessed with a unpaired two-tailed Student's *t*-test unless otherwise specified and was accepted at the *P*<0.05 level. The data distributions were assumed to be normal, but this was not formally tested.
